# Differential Expression of Host Biomarkers in Saliva and Serum Samples from Individuals with Suspected Pulmonary Tuberculosis

**DOI:** 10.1155/2013/981984

**Published:** 2013-11-13

**Authors:** Khutso G. Phalane, Magdalena Kriel, Andre G. Loxton, Angela Menezes, Kim Stanley, Gian D. van der Spuy, Gerhard Walzl, Novel N. Chegou

**Affiliations:** DST/NRF Centre of Excellence for Biomedical Tuberculosis Research and MRC Centre for Molecular and Cellular Biology, Division of Molecular Biology and Human Genetics, Department of Biomedical Sciences, Faculty of Medicine and Health Sciences, University of Stellenbosch, P.O. Box 19063, Francie van Zijl Drive, Tygerberg 7505, South Africa

## Abstract

The diagnosis of tuberculosis remains challenging in individuals with difficulty in providing good quality sputum samples such as children. Host biosignatures of inflammatory markers may be valuable in such cases, especially if they are based on more easily obtainable samples such as saliva. To explore the potential of saliva as an alternative sample in tuberculosis diagnostic/biomarker investigations, we evaluated the levels of 33 host markers in saliva samples from individuals presenting with pulmonary tuberculosis symptoms and compared them to those obtained in serum. Of the 38 individuals included in the study, tuberculosis disease was confirmed in 11 (28.9%) by sputum culture. In both the tuberculosis cases and noncases, the levels of most markers were above the minimum detectable limit in both sample types, but there was no consistent pattern regarding the ratio of markers in serum/saliva. Fractalkine, IL-17, IL-6, IL-9, MIP-1**β**, CRP, VEGF, and IL-5 levels in saliva and IL-6, IL-2, SAP, and SAA levels in serum were significantly higher in tuberculosis patients (*P* < 0.05). These preliminary data indicate that there are significant differences in the levels of host markers expressed in saliva in comparison to those expressed in serum and that inflammatory markers in both sample types are potential diagnostic candidates for tuberculosis disease.

## 1. Introduction

Tuberculosis (TB) remains a global health problem. An estimated 8.7 million new cases and 1.4 million deaths resulted from the disease in 2011 [1]. A delay or failure in the diagnosis of the disease results in treatment delay with consequent increased opportunity for transmission, with potentially ten people infected annually per untreated case [2]. The low sensitivity of smear microscopy, the most commonly used TB diagnostic test in resource-constrained settings, is well-publicized [3, 4]. *Mycobacterium tuberculosis* (*M.tb*) culture facilities are not widely available in resource-limited settings and culture results may take up to 42 days to become available [5]. The development of the Xpert MTB/RIF test (Cepheid Inc., CA, USA) has been the most important advance in the field as the test yields results within 2 hours coupled with the detection of rifampicin resistance [6]. The test has a pooled sensitivity of 67–98% and specificity of about 98% in adults [7]. However, limitations, including high costs and the requirement for a stable electricity supply and short shelf life of consumables [8], hamper the massive roll-out of the test in resource-constrained and often high-burden settings. Furthermore, diagnostic tests based on sputum are not suitable in individuals who have difficulty in providing good quality sputum samples such as children [9] and those with extrapulmonary TB disease. Immunodiagnostic techniques employing host biosignatures of inflammatory markers could be valuable in such cases [10, 11], especially if based on more easily obtainable samples such as saliva and developed into rapid point-of-care tests.

Saliva is primarily secreted through the parotid, submandibular, and sublingual glands. It is composed of 98% water and contains other substances including electrolytes, mucus, antibacterial compounds, and various enzymes [12]. It is abundantly produced in individuals of all age groups and an average human produces 0.3 to 7 mL of saliva per minute and always has about 1 mL in the oral cavity [12]. Collection of saliva is simple, is noninvasive, and does not carry the inconveniences or risks of drawing blood [13]. 

There has recently been an interest in exploring saliva for potentially useful inflammatory biomarkers [13]. Diagnostic tests based on saliva, such as the HIV oral fluid rapid tests [14], are commercially available. Despite the large number of TB biomarker discovery studies which are available in the literature, most of which are based on serum or, to a lesser extent, urine [15–18] (reviewed in [19–21]), saliva, a relatively easy-to-obtain and abundant sample type, has not yet attracted much interest in the field. In the present study, we assessed the levels of 33 host markers in saliva of individuals presenting with symptoms suggestive of pulmonary TB and compared them to the levels detected in serum. We show that there are large differences in the levels of markers expressed in saliva in comparison to serum and that some of the salivary markers may have potential in the diagnosis of TB disease. 

## 2. Materials and Methods

### 2.1. Study Participants

Individuals suspected of having pulmonary TB disease were recruited from the Fisantekraal community in the outskirts of Cape Town, South Africa, as part of the ongoing EDCTP-funded African European Tuberculosis Consortium (AE-TBC) study (http://www.ae-tbc.eu/). Recruitment of study participants began in October 2010. At the time this pilot study was conducted, 50 participants had been enrolled at the study site, and pulmonary TB disease had been confirmed in 11 (22%). Samples from all the 11 individuals with confirmed TB disease and 27 without TB disease that were randomly selected from our sample bank were included into this preliminary study. All the individuals included in the study provided the sample pair (saliva and serum).

All participants presented at the rural health care facility with symptoms suggestive of pulmonary TB disease. Briefly, participants presented with persistent cough lasting ≥2 weeks and at least one of the following: fever, malaise, recent weight loss, night sweats, knowledge of close contact with a TB patient, haemoptysis, chest pain, or loss of appetite. Participants were eligible for the study if they were 18 years or older, willing to give written informed consent for participation in the study, including for HIV testing. Patients were excluded from the study if they had not been residing in the study area for more than 3 months, were severely anaemic (HB < 10 g/L), were on anti-TB treatment, had received anti-TB treatment in the previous 90 days, or were on quinolone or aminoglycoside antibiotics in the past 60 days. At enrolment, a case report form was completed for each participant before blood and saliva samples along with other samples, including urine and sputum as required for the main study, were collected. The study was approved by the Health Research Ethics Committee of the University of Stellenbosch (Reference no. N10/08/274) and the City of Cape Town.

### 2.2. Sample Collection and Diagnostic Tests

Blood was collected into 4 mL plain BD vacutainer tubes (BD Biosciences) and transported at ambient conditions to the laboratory. The tubes were then centrifuged at 2500 rpm for 10 minutes after which serum was harvested, aliquoted, and frozen (−80°C) until use. Saliva was collected from all participants into salivette tubes (Sarstedt, Nümbrecht, Germany), according to the instructions of the manufacturer. Saliva samples were then transported on ice (4°C) to the laboratory after which the salivette tubes were centrifuged for 2 minutes (1000 g) and the saliva was harvested, aliquoted into labeled tubes, and kept at −80°C until analysis.

Sputum samples collected from all participants were cultured by the MGIT method (BD Biosciences). Positive MGIT samples were examined for AFB using the Ziehl-Neelsen method, to check for contamination, after which PCR experiments were performed to confirm the isolation of* M.tb* complex organisms. All the individuals classified as TB cases in this study had positive *M.tb* complex speciated sputum cultures and other clinical features in keeping with TB, including typical chest X-rays. The individuals in the “No TB” group had negative sputum smears and cultures and had no other signs suggestive of TB including negative chest X-rays. None of the non-TB cases was treated for TB by the national TB control program. All sputum and saliva samples were processed in a BSL3 laboratory.

### 2.3. Luminex Multiplex Immunoassay

The levels of 33 host markers, including interferon (IFN)-*γ*, interleukin (IL)-1*β*, IL-1*α*, IL-2, IL-4, IL-5, IL-6, IL-7, IL-8, IL-9, IL-10, IL-12(p70), IL-13, IL-15, IL-17, soluble IL-2 receptor alpha (sIL-2R*α*), interferon inducible protein (IP)-10, tumor necrosis factor (TNF)-*α*, fractalkine, granulocyte monocyte colony stimulating factor (GM-CSF), epidermal growth factor (EGF), monocyte chemotactic protein (MCP)-1, macrophage inflammatory protein (MIP)-1*β*, soluble CD40 ligand (sCD40L), transforming growth factor (TGF)-*α*, vascular endothelial growth factor (VEGF), granulocyte colony stimulating factor (G-CSF), CXCL1(GRO), C-reactive protein (CRP), serum amyloid protein A (SAA), serum amyloid protein P (SAP), matrix metalloproteinase (MMP)-2, and MMP-9, were evaluated in serum and saliva samples from all study participants. This was done using customized Milliplex kits (Merck Millipore, St. Charles, Missouri, USA) on the Bio-Plex platform (Bio-Rad Laboratories, Hercules, CA, USA). All samples were analyzed undiluted (with the exception of the samples for MMP-2, MMP-9, CRP, SAA, and SAP) and in a blinded manner, according to the instructions of the manufacturer (Merck Millipore). Samples for MMP-2 and MMP-9 were prediluted 1 : 100 and those for CRP, SAA, and SAP were prediluted 1 : 8000, following optimization experiments (done on serum). Serum and saliva samples from the same individual were evaluated on the same plate. The levels of all analytes in the quality control reagents were within the expected ranges. The Bio-Plex Manager Software 6.0 was used for bead acquisition and analysis of median fluorescence intensity.

### 2.4. Statistical Analysis

Differences in analyte levels between the TB patients and participants without TB disease or between the marker levels detected in saliva and serum levels were evaluated by the Mann-Whitney *U* test for nonparametric data analysis. The diagnostic accuracy of the markers was investigated by receiver operator characteristics (ROC) curve analysis. Optimal cut-off values and associated sensitivity and specificity were selected based on the highest likelihood ratio. The predictive abilities of combinations of analytes were estimated by performing best subsets general discriminant analysis (GDA), with leave-one-out cross validation. Nonnormally distributed data were log-transformed prior to the GDA procedure. Differences between groups were considered significant if *P* values were <0.05. Data were analyzed using GraphPad Prism, version 5.00 (GraphPad Software, San Diego, California, USA) and STATISTICA (StatSoft, Ohio, USA).

## 3. Results

Of the 38 participants included in this study, 27 (71%) were females. The mean age of all study participants was 38.0 ± 10.2. Of the 28 study participants with available Quantiferon In-Tube results, 67.9% were positive using the manufacturer's recommended cut-off (≥0.35 IU/mL). Eight (21%) of the study participants were HIV infected (Table 1).

### 3.1. Host Markers Detected in Saliva versus Serum

We evaluated the levels of host markers above the minimum detectable concentration (MDC; obtained from the manufacturer's package insert), in saliva and serum, and then compared the levels of the markers detected in saliva to those obtained in serum. The levels of five of the 33 markers evaluated (MMP-9, IP-10, MIP-1*β*, MCP-1, and sCD40L) were higher than the MDC, in both the serum and saliva samples from all (100%) of the study participants. The levels of six (IL-8, G-CSF, TGF-*α*, EGF, VEGF, and GRO) were above the MDC in both sample types in at least 90% of the study participants, while the levels of IL-4 and IL-9 were undetectable or only barely detectable in both sample types, in all study participants (Table 2). 

**Figure 1:**
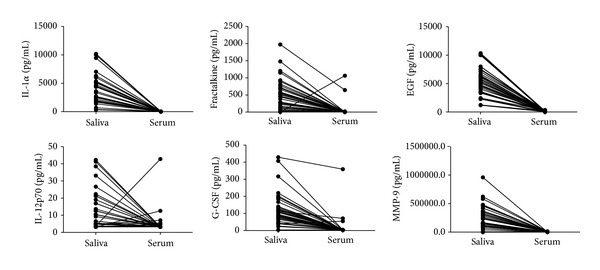
Levels of host markers detected in saliva and serum samples from all study participants (*n* = 38). The level of each host marker detected in saliva was mapped to the level obtained in the serum sample from the same study participant. Representative plots for markers more abundantly expressed in saliva are shown.

**Figure 2:**
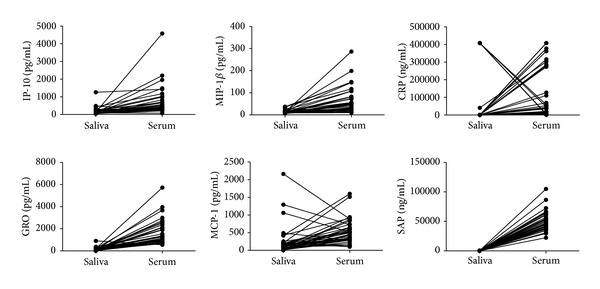
Levels of host markers detected in saliva and serum samples from all study participants (*n* = 38). The level of each host marker detected in Saliva was mapped to the level obtained in the serum sample from the same study participant. Representative plots for markers more abundantly expressed in serum are shown.

There were, on average, 6-fold higher levels of IFN-*γ*, IL-1*α*, IL-12(p70), IL-13, IL-15, IL-17, fractalkine, GM-CSF, and EGF in saliva samples from all study participants in comparison to serum levels. The levels of IL-1*β*, IL-2, IL-5, IL-6, IL-7, IL-8, IL-10, G-CSF, VEGF, and MMP-9 were also significantly higher in saliva (Table 2, Figure 1). There were, on average, 4-fold higher levels of IP-10, MIP-1*β*, GRO, and CRP detected in serum samples of all study participants in comparison to the salivary levels. SAA, SAP, sIL-2R*α*, sCD40L, MCP-1, and MMP-2 levels were also significantly higher in serum (Table 2, Figure 2). When the marker levels obtained in serum were compared to the levels obtained in saliva only in the TB or non-TB cases, the same expression pattern (obtained for all study participants, that is, the two groups together) was observed (data not shown).

### 3.2. Accuracy of Markers Detected in Saliva in the Diagnosis of TB Disease

**Figure 3:**
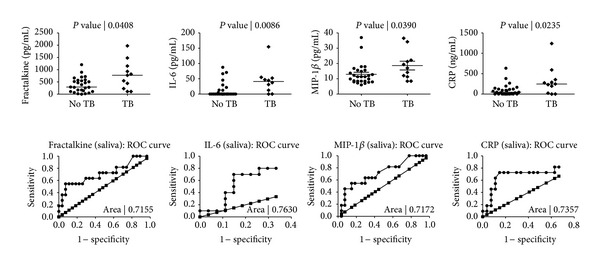
Levels of markers detected in the saliva samples of pulmonary TB cases and individuals without TB disease and receiver operator characteristics (ROC) plots showing the accuracy of these markers in the diagnosis of TB disease. Error bars in the scatter-dot plots indicate the median analyte levels. Only markers for which the area under the ROC curve (AUC) was ≥0.70 are shown.

When the levels of markers detected in saliva of TB cases were compared to the levels obtained in the saliva of the noncases, 8 of the 33 markers (IL-6, CRP, IL-9, IL-5, MIP-1*β*, fractalkine, IL-17, and VEGF) were significantly different, or showed trends for differences between the two groups (Table 3). With the exception of VEGF, the median levels of all 8 markers were higher in the TB cases (Table 3). When the diagnostic accuracy of the markers was evaluated by receiver operator characteristics (ROC) curve analysis, IL-6, CRP, MIP-1*β*, and fractalkine showed potential in the diagnosis of TB disease (AUC ≥ 0.70) (Table 3). Although none of the markers diagnosed TB disease with sensitivity ≥64% at the cut-off level corresponding to the highest likelihood ratio, salivary CRP, MIP-1*β*, and fractalkine levels ascertained TB disease with specificity ≥93% (Table 3, Figure 3).

**Figure 4:**
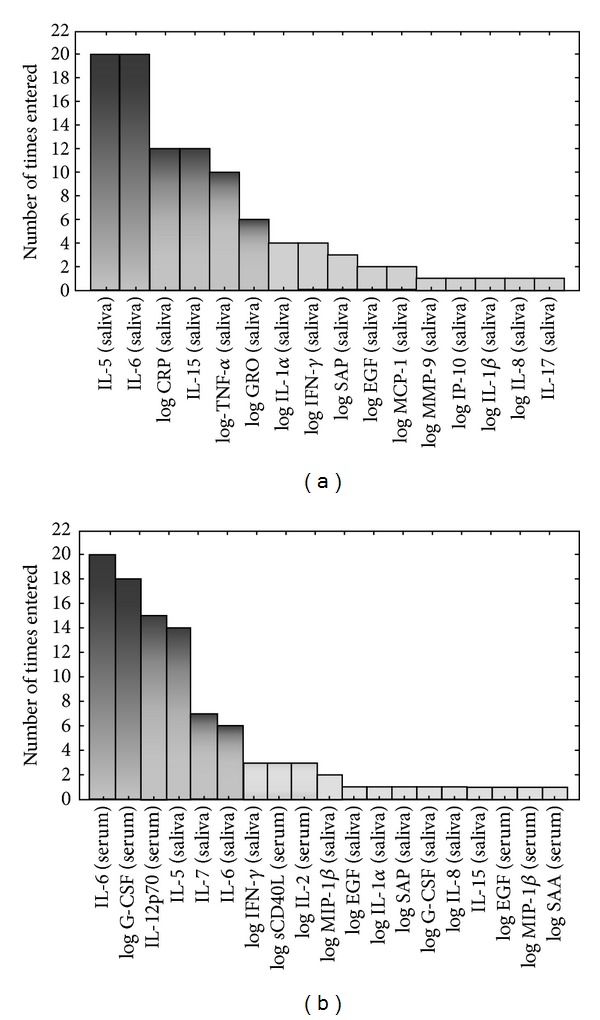
Frequency of analytes in the top 20 GDA predictive models that most accurately classified study participants as TB disease or no TB. The columns represent the number of times each analyte was included in the top 20 discriminatory models. (a) Frequency of analytes in the models generated from the host markers detected in saliva, (b) frequency of analytes in models generated when the data obtained from saliva were combined with the data obtained from serum samples.

When the data obtained from saliva were fitted into general discriminant analysis (GDA) models, optimal prediction of TB or no TB disease was achieved when markers were used in combinations of five. A combination of IL-5, IL-6, IL-15, TNF-*α*, and CRP accurately predicted 81.8% of the TB cases and 81.4% of the noncases after leave-one-out cross validation. The most frequently occurring analytes in the 20 most accurate discriminatory models included IL-5, IL-6, IL-15, CRP, TNF-*α*, and GRO (Figure 4). 

### 3.3. Accuracy of Markers Detected in Serum in the Diagnosis of TB Disease

When serum marker levels obtained in TB cases were compared to the levels obtained in the noncases, significant differences were obtained for four markers (IL-6, IL-2, SAP, and SAA). The levels of IL-6, IL-2, and SAP were significantly higher in the TB cases (*P* ≤ 0.03) while SAA levels were higher in the noncases (Table 4).

**Figure 5:**
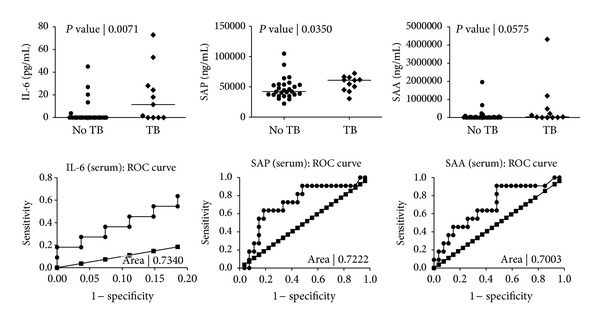
Levels of markers detected in the serum samples of pulmonary TB cases and individuals without TB disease and receiver operator characteristics (ROC) plots showing the accuracy of these markers in the diagnosis of TB disease. Error bars in the scatter-dot plots indicate the median analyte levels. Only markers for which the area under the ROC curve (AUC) was ≥0.70 are shown.

When the diagnostic accuracy of the markers detected in serum was investigated by ROC curve analysis, the AUC for all four markers that showed significant differences (IL-2, IL-6, SAP, and SAP) was ≥0.70 (Table 4, Figure 4). Of the four markers, only SAP ascertained TB disease with sensitivity up to 55%, but specificity was between 85.2% and 96.3% for all four markers at cut-off levels corresponding to the highest likelihood ratio (Table 4, Figure 5).

When the data obtained from serum samples was fitted into GDA models, the prediction accuracy of the 5-marker serum analytes tended to be poorer than that obtained for the models generated on saliva data. A combination of IL-6, IL-12p70, G-CSF, MMP-9, and MIP-1*β* could only predict 54.6% of the TB cases and 81.5% of the noncases after leave-one-out cross validation. When the GDA procedure was repeated, with the data obtained from serum and saliva combined, the prediction accuracy of the models increased. IL-5 and IL-6 levels in saliva + G-CSF, IL-12p70, and IL-6 in serum accurately classified all (100%) of the TB cases and 85.2% of the noncases after leave-one-out cross validation. The most frequently occurring analytes in the predictive models comprising of markers derived from both serum and saliva included serum IL-6, G-CSF, and IL-12p70 and salivary IL-5, IL-6, and IL-7 (Figure 4).

## 4. Discussion

We evaluated the levels of 33 host immunological biomarkers in saliva and serum samples from individuals suspected of having pulmonary TB disease. The main finding of our study was the dissimilar expression of host markers that were detected in both sample types, with up to 6-fold higher levels of some markers expressed in saliva. We have therefore shown that saliva, a relatively easy-to-obtain sample type, may be a very valuable sample in TB biomarker discovery investigations. Interestingly, some of the markers detected in saliva including IL-6, CRP, MIP-1*β*, and fractalkine showed potential in the diagnosis of TB disease.

All the markers evaluated in this study are inflammatory markers including cytokines, chemokines, growth factors, acute phase proteins, and matrix metalloproteinases that have been widely investigated and shown to play diverse roles in the pathogenesis of different diseases including TB. Of note, the levels of IL-5, IL-6, IL-9, IL-17, MIP-1*β*, fractalkine, CRP, and VEGF in saliva and those of IL-2, IL-6, SAA, and SAP in serum were significantly different or showed trends (*P* ≤ 0.009) between the TB cases and individuals without active TB disease. IL-2, IL-6, MIP-1*β*, fractalkine, CRP, SAA, and SAP in serum or saliva showed potential as TB diagnostic candidates, as ascertained by area under the ROC curve. 

IL-2 (reviewed in [22]) is an important immunomodulatory cytokine that is produced by multiple cell types including activated T-cells, dendritic cells, and NK cells and is crucial both for the immune responses against many infectious diseases and for the maintenance of tolerance [22]. IL-2 has been extensively investigated especially in T-cell based studies and has shown potential as a diagnostic marker for TB disease [23, 24]. IL-6 (reviewed in [25]) is a pleiotropic cytokine and has diverse effects on the regulation of immune responses, inflammation, oncogenesis, and haematopoiesis amongst others and is widely known as an inducer of the acute phase response. IL-6 has previously been shown to be produced in higher levels in TB patients [26, 27] and was the only marker that showed potential as a diagnostic candidate in both serum and saliva samples in this study. Fractalkine is induced in endothelial cells and antigen presenting cells in the presence of various TH1 favoring signals including IFN-*γ*, CD40L, and TNF-*α* and is inhibited in the presence of IL-4/IL-13 [28]. MIP-1*β* is mainly produced by macrophages, dendritic cells, NK cells, and T-cells [29, 30]. It has been investigated as a diagnostic candidate in T cell based studies, but the potential shown in some adult studies [31, 32] was not confirmed in children [33]. In this study, MIP-1*β* showed potential in the diagnosis of TB disease in saliva but not in serum. CRP, SAA, and SAP are acute phase proteins and are secreted during the acute phase of an inflammation where they function as opsonins or in the recruitment of cells to inflammatory sites [34]. These nonspecific inflammatory markers are known to be predominantly produced by the liver and have been investigated in many diseases [34, 35]. CRP has previously been suggested as a marker for both the diagnosis [35, 36] and extent of disease in TB [37, 38]. SAA has been suggested as a more sensitive indicator of inflammation than CRP [39], and serum levels increase by 1000-fold in response to infection [40]. Like SAA, SAP is structurally similar to CRP (51% sequence homology) and SAP levels have been proved to be high in TB in murine models, whereas purified mouse SAP inhibited the growth of *M.tb* in alveolar macrophages [41]. Our observations that these markers possess diagnostic potential for TB disease are in line with these previous findings.

One of the most noticeable observations of our study was the marked differences in the levels of the markers detected in saliva and serum and that higher levels of most of the markers were obtained in saliva. Most of the markers that were more abundantly expressed in serum were chemokines (IP-10, MIP-1*β*, and MCP-1), acute phase proteins (CRP, SAA, SAP), and sIL-2R and sCD40L, both are ligands for molecules required for activation and differentiation of T cells, and GRO is an angiogenesis mediator. Higher levels of the most commonly investigated proinflammatory cytokines and growth factors were obtained in saliva. Our observations are in agreement with findings that the salivary gland is a reservoir of many growth factors in rodents [42]. In humans, EGF, basic fibroblast growth factor, insulin, and insulin-like growth factor family members have been detected in the salivary gland, even though their physiological role remains unclear [42].

Although investigations on saliva in the TB biomarker field are limited, saliva has been widely investigated in other diseases, notably in leukaemia, oral cancer, oral lichen planus, and periodontitis amongst others [43, 44]. US FDA approved saliva-based commercial HIV rapid tests which currently exist [45]. The many advantages of using saliva as a diagnostic sample have been discussed elsewhere [44]. Our observations confirm the potential usefulness of saliva in TB biomarker research. The high levels of the host markers in saliva implies that these markers may be more reliably measured even with lateral flow devices, which are usually easy to perform and suitable even for remote settings, but often have low limits of detection. Although diagnostic tests based on salivary inflammatory markers may lack specificity as the levels of these markers might also be high in other diseases, the markers might be useful when combined with clinical information. Such tests will be highly useful in the TB diagnostic field, given the difficulties obtained with diagnosing TB disease when inadequate sputum is obtained and when there is an inability to expectorate as obtained in young children [9] and in extrapulmonary TB cases. All the TB cases evaluated in this study were adults and all were diagnosed with pulmonary TB diseases. We cannot ascertain, based on the data presented in this study, whether these markers will be useful in children and in those with extrapulmonary TB disease. There is therefore a need for further investigations in children, those with extra pulmonary TB and as those with other lung diseases. Children and those with extrapulmonary TB in particular would benefit the most from novel, easy-to-perform nonsputum based diagnostic tests. Although the use of a biosignature comprising inflammatory cytokines is highly desirable and needed in the TB diagnostic filed, it is important to caution that most of the markers identified in this study (e.g., the acute phase proteins) will be elevated in other infectious diseases. Therefore, data obtained from such diagnostic modalities will need to be interpreted, taking into account the clinical picture of the patient. 

This study was done as a pilot for a larger, on-going study and, as such, was limited by the small sample size and the absence of individuals with other lung infections. However, our observations will serve as proof-of-concept for more research in the field given the fact that diagnostic tests based on easily obtainable samples like saliva would revolutionize the diagnosis of TB disease, especially if such markers are incorporated into lateral flow devices. We did not investigate the influence of factors such as food or drink in-take prior to sampling on the levels of the salivary biomarkers. The potential influence of such factors may require investigation in further studies.

## 5. Conclusions

In conclusion, the data presented in this study indicates that there are many differences in the levels of host markers expressed in saliva in comparison to those of serum and some of the markers detected in both sample types have potential in the diagnosis of TB disease. Our findings indicate that saliva might be a better alternative to serum in TB biomarker discovery investigations. Our findings warrant further investigation in larger studies.

## Figures and Tables

**Table 1:** Demographic and clinical characteristics of study participants.

	All	TB cases	Non-TB case
Number of participants	38	11	27
Mean age (years) ± SD	38.0 ± 10.2	43.4 ± 8.7	35.9 ± 10.1
Males/females	11/27	3/8	8/19
HIV positive *n* (%)	8 (21.0)	2 (18.2)	6 (22.2)
Quantiferon Positive *n*/number done	19/28	5/6	14/22

**Table 2:** Proportion of study participants with host markers above the minimum detectable concentration (MDC) and differences between saliva and serum.

Marker	MDC (pg/mL)	Saliva		Serum		*P* value
		% >MDC	Median (IQR)	% >MDC	Median (IQR)	
(A) Host markers more abundantly expressed in saliva						
IL-1α	1.5	100.0	4618.9 (1956.3–10000.0)	21.0	0.0 (0.0-0.0)	<0.0001
IL-1β	0.7	95.0	24.6 (12.23–54.7)	18.0	0.0 (0.0–0.2)	<0.0001
IL-2	0.4	97.0	6.5 (2.3–14.4)	32.0	0.0 (0.0–0.6)	<0.0001
IL-5	0.1	32.0	0.0 (0.0–1.1)	0.0	0.0 (0.0-0.0)	<0.0001
IL-7	1.0	45.0	0.0 (0.0–19.0)	16.0	0.0 (0.0-0.0)	<0.0001
IL-8	0.3	100.0	145.2 (78.6–237.3)	97.0	13.6 (6.2–27.7)	<0.0001
IL-10	0.3	39.5	0.0 (0.0–16.8)	2.6	0.0 (0.0-0.0)	<0.0001
IL-12p70	0.9	89.0	9.8 (3.7–16.9)	16.0	0.0 (0.0–0.3)	<0.0001
IL-13	0.3	92.0	20.7 (11.4–34.1)	0.0	0.0 (0.0-0.0)	<0.0001
IL-15	0.6	45.0	0.0 (0.0–8.4)	5.0	0.0 (0.0-0.0)	<0.0001
IL-17	0.4	97.0	13.0 (8.6–18.9)	16.0	0.0 (0.0-0.0)	<0.0001
IFN-*γ*	0.4	82.0	4.1 (0.6–10.4)	42.0	0.0 (0.0–5.0)	<0.0001
G-CSF	3.9	100.0	1348.0 (842.0–2263.2)	97.0	90.7 (45.3–114.4)	<0.0001
GM-CSF	2.3	100.0	100.5 (65.3–137.9)	8.0	0.0 (0.0-0.0)	<0.0001
TGF-α	1.4	100.0	9.5 (6.7–16.6)	92.0	6.9 (3.2–20.8)	0.08
EGF	5.3	100.0	5717.0 (3991.9–7964.4)	97.0	98.6 (45.5–185.1)	<0.0001
VEGF	10.1	100.0	618.2 (457.3–802.6)	95.0	303.5 (145.7–493.2)	<0.0001
Fractalkine	7.6	97.0	451.8 (137.9–699.9)	10.0	0.0 (0.0-0.0)	<0.0001
MMP-9	1.0	100.0	164631.4 (105484.3–348292.9)	100.0	2673.0 (1795.9–3951.6)	<0.0001

(B) Markers more abundantly expressed in serum						
sIL-2R*α*	7.5	8.0	0.0 (0.0-0.0)	29.0	0.0 (0.0–10.4)	0.01
GRO	11.4	97.0	132.5 (74.1–204.0)	100.0	1209.1 (856.0–2099.6)	<0.0001
IP-10	1.3	100.0	102.6 (67.4–213.3)	100.0	408.0 (307.4–710.0)	<0.0001
MIP-1*β*	3.2	100.0	12.0 (8.4–17.0)	100.0	47.7 (22.2–81.3)	<0.0001
MCP-1	1.2	100.0	124.5 (29.5–204.0)	100.0	473.4 (314.3–644.6)	<0.0001
CRP	0.0012*	71.0	88.2 (0.0–232.0)	100.0	27668.5 (9213.7–127253.2)	<0.0001
SAA	0.21*	50.0	119.5 (0.0–848.8)	97.0	11408.9 (2519.1–95050.4)	<0.0001
SAP	0.055*	21.0	0.0 (0.0-0.0)	100.0	46954.9 (37567.1–60894.8)	<0.0001
MMP-2	48	13.0	0.0 (0.0-0.0)	100.0	1148.8 (971.1–1333.0)	<0.0001
sCD40L	5.2	100.0	353.8 (166.9–779.2)	100.0	*705911 (307862–1039000)	<0.0001

(C) No difference in expression levels between serum and saliva						
IL-4	0.6	0.0	0.0 (0.0-0.0)	0.0	0.0 (0.0-0.0)	—
IL-6	0.4	45.0	0.0 (0.0–37.3)	32.0	0.0 (0.0–11.5)	0.12
IL-9	1.1	15.8	0.0 (0.0-0.0)	5.3	0.0 (0.0-0.0)	0.14
TNF-*α*	0.2	87.0	10.6 (6.9–20.6)	95.0	11.1 (6.5–13.0)	0.23

Median levels of biomarkers detected in saliva and serum samples from all study participants (n = 38) and the proportion of participants in whom each marker was >MDC are shown. MDC values were obtained from the package insert of the kit used. *Marker levels are in pg/mL except for CRP, SAA, and SAP (ng/mL). IQR: inter-quartile range.

**Table 3:** Utility of host markers detected in saliva in the diagnosis of TB disease.

Marker	TB disease	No TB disease	*P* value	AUC (95% CI)	Cut-off value	Sensitivity % (95% CI)	Specificity % (95% CI)
IL-6	37.3 (0.0–52.2)	0.0 (0.0–13.2)	0.019	0.72 (0.54–0.91)	>25.8	63.6 (30.8–89.0)	81.5 (61.9–93.7)
CRP	246.5 (22.0–353.9)	45.9 (0.0–122.0)	0.024	0.74 (0.53–0.94)	>271.7	45.5 (16.8–76.6)	92.6 (75.7–99.0)
IL-9	0.0 (0.0–11.0)	0.0 (0.0-0.0)	0.027	0.65 (0.44–0.86)	>10.3	27.3 (6.0–60.9)	96.3 (81.0–99.9)
IL-5	0.9 (0.0–9.2)	0.0 (0.0-0.0)	0.033	0.68 (0.48–0.88)	>7.8	27.3 (6.0–61.0)	96.3 (81.0–99.9)
MIP-1*β*	17.0 (11.3–22.2)	11.3 (8.4–15.6)	0.039	0.72 (0.54–0.90)	>18.7	45.5 (16.8–76.6)	92.6 (75.7–99.1)
Fractalkine	772.9 (225.8–1148.3)	338.2 (104.3–565.5)	0.041	0.72 (0.52–0.91)	>912.2	36.4 (10.9–69.2)	96.3 (81.0–99.9)
IL-17	18.9 (7.6–37.0)	12.6 (8.6–16.6)	0.085	0.68 (0.46–0.90)	>29.0	45.5 (16.8–76.6)	96.3 (81.0–99.9)
VEGF	457.3 (307.7–754.9)	680.0 (512.4–802.6)	0.085	0.68 (0.47–0.90)	<370.5	45.5 (16.8–76.6)	92.6 (75.7–99.1)

Median levels and interquartile ranges (in parenthesis) of the markers and abilities to discriminate between pulmonary TB cases (*n* = 11) and individuals without active TB (*n* = 27) are shown. Only markers for which Mann-Whitney *U* *P* values were ≤0.09 are shown. AUC: area under the receiver operator characteristics (ROC) curve; 95% CI: 95% confidence interval. The cut-off values are for sensitivity and specificity for TB disease and were selected based on the highest likelihood ratio. Marker levels are in pg/mL except for CRP (ng/mL).

**Table 4:** Utility of host markers detected in serum in the diagnosis of TB disease.

Marker	TB cases	Non-TB cases	*P* value	AUC (95% CI)	Cut-off value	Sensitivity % (95% CI)	Specificity % (95% CI)
IL-6	11.5 (0.0–28.1)	0.0 (0.0-0.0)	0.01	0.73 (0.54–0.92)	>27.54	27.3 (6.0–61.0)	96.3 (81.0–99.9)
IL-2	0.6 (0.0–1.3)	0.0 (0.0-0.0)	0.01	0.73 (0.53–0.92)	>0.95	45.5 (16.8–76.6)	92.6 (75.7–99.1)
SAP	60894.8 (45137.4–65623.2)	42251.4 (36985.9–53804.8)	0.03	0.72 (0.54–0.90)	>58914	54.6 (23.4–83.3)	85.2 (66.3–95.8)
SAA	239.1 (0.0–848.8)	6133.8 (2012.1–40070.2)	0.05	0.70 (0.52–0.88)	>941894	18.2 (2.3–51.8)	96.3 (81.0–99.9)

Median levels (pg/mL) and interquartile ranges (in parenthesis) of the markers and abilities to discriminate between pulmonary TB cases (n = 11) and individuals without active TB (n = 27) are shown. Only markers for which Mann-Whitney *U* *P* values were ≤0.09 are shown. AUC: area under the receiver operator characteristics (ROC) curve; 95% CI: 95% confidence interval. The cut-off values are for sensitivity and specificity for TB disease and were selected based on the highest likelihood ratio. Marker levels are in pg/mL except for SAA and SAP (ng/mL).
